# Clinical significance of microRNA-1180-3p for colorectal cancer and effect of its alteration on cell function

**DOI:** 10.1080/21655979.2021.1997694

**Published:** 2021-12-11

**Authors:** Chunlei Li, Wei Jin, Dewei Zhang, Shoujin Tian

**Affiliations:** a Department of Gastroenterology, Jiaozhou Central Hospital of Qingdao, Shandong, China; b Department of Gastroenterology, Yidu Central Hospital of Weifang, Shandong, China; c Department of 3rd General Surgery, The Fourth Affiliated Hospital of China Medical University, Liaoning, China; d Department of General Gastroenterology, Zhucheng People’s Hospital, Shandong, China

**Keywords:** Colorectal cancer, miR-1180-3p, prognosis, growth, mobility

## Abstract

An early diagnosis and effective prognostic factors would greatly reduce the mortality rate of colorectal cancer (CRC). This research is intended to complete the evaluation of the prognostic value and potential role of miR-1180-3p in CRC. The miR-1180-3p levels were reduced in CRC patients’ tissues, blood, and human CRC cell lines. The ability of miR-1180-3p was explored in discrimination of CRC patients and healths and the value in overall survival estimate. The effect of miR-1180-3p dysregulation on the CRC cellular function was investigated. miR-1180-3p is downregulated in CRC tissues, blood and cells than normal ones. This lower expression was correlated with vascular invasion, lymph node metastasis, and TNM stage. With the use of ROC curve, miR-1180-3p showed discriminating ability in CRC patients and healthy subjects. With the result of Kaplan–Meier analysis and multi-multivariate Cox analysis, miR-1180-3p was an independent predictor for CRC patients’ overall survival. Utilizing CCK-8, Transwell and matrigel assays, overexpression of miR-1180-3p reduced cancer cell proliferation and mobility, but induced apoptosis, by targeting *COL12A1*. miR-1180-3p might function as a suppressor in CRC progression and allowed the discovery of a new biomarker for diagnosis, prognosis and therapy target for CRC.

## Introduction

Colorectal cancer (CRC) has come to the fourth in all types of cancers worldwide and ranks third most diagnosed and fifth lethal cancer in China [1,2]. According to the WHO Incidence Database, approximately 2 million new CRC patients were diagnosed in 2020, and the estimated number of deaths in 2020 due to CRC is more than nine hundred thousand [3,4]. What is more serious, the upward trend of CRC in China was obvious for age-specific incidence rate, which exists in the age group of 40–59 years for both males and females [2,5]. The current screening strategies for CRC include fecal occult blood tests and invasive endoscopy-based investigations for making a diagnosis [6]. However, the positive-predictive value of the hemoglobin concentration in fecal ranged from 21% to 76%, resulting in many false positives and additional diagnostic evaluations [7]. Colonoscopy can miss lesions and its invasive procedure easily caused the public fears [8]. In addition, the 5-year survival relative rate for CRC patients was unsatisfactory with a range between 90% and 14% [9]. Thus, new strategies that are efficacious and practical are indeed needed to complement or revolutionize CRC screening and prognostic prediction.

Epigenetic markers could be used in the screening of cancers or prediction of disease progression [10]. Epigenetic changes include the alterations of mRNAs translation through interacting with non-coding RNAs, including microRNAs (miRNAs) [11,12]. Though without the ability to transcribe into proteins, miRNAs regulate gene expression at both the transcriptional and post-transcriptional levels [13]. Alternations in miRNA levels and activities have been observed in many cancer cells [14]. Benefited from the small size and hairpin-loop structure, microRNAs are protected from degradation and their levels might be used as diagnostic and prognostic markers of cancer [15]. In addition, miRNAs are usually secreted by tumors into the circulation or gastrointestinal tract, which makes it feasible to extract miRNAs from specimens such as blood [16,17]. For instance, a significant decrease of miR-1207-5p in CRC plasma can predict poor survival and showed strong diagnostic ability for CRC [18]. Moreover, previous studies found that miRNAs played important roles in the progression of cancers, such as ovarian cancer [19], hepatocellular carcinoma [20]. miR-1180-3p has been reported with distinguishing ability between early gastric cancer and the healthy control group [21] and was identified as a dysregulated miRNA in serum from CRC patients [22]. But the value of miR-1180-3p in CRC diagnosis and prognosis has not been tested and verified.

In this forthcoming study, miR-1180-3p was hypothesized as a potential diagnostic and prognostic factor for CRC, and an important regulator in cancer progression. First, the expression of miR-1180-3p was determined in CRC samples (tissues, blood) and cells to find whether it shows an aberrant expression level or not. Then, based on aberrant expression level, the diagnostic value and survival-estimated value were evaluated to verify the clinical significance of miR-1180-3p. Apart from these, cell function experiments were performed to access whether miR-1180-3p is involved in the progression of CRC.

## Material and methods

### Patients, tissue, and blood specimens

This study comprised 134 CRC tissues and serum samples, and 104 healthy serum specimens from healthy volunteers. All subjects signed written informed consent. This study was carried out according to the approved guidelines by the Institutional Research Ethics Committee of Jiaozhou Central Hospital of Qingdao (no. 201,002).

The diagnostic criteria for CRC patients have followed the stage method of the American Joint Committee on Cancer staging and the International Union against Cancer TNM staging system [23]. The CRC patients, who were new diagnostic and received no treatment concerning CRC before surgical resection were screened from Jiaozhou Central Hospital of Qingdao between March 2010 and March 2016. The complete medical documents were able to be transferred. The healthy volunteers who had no prior diagnosis of any other tumor served as normal controls.

### RNA isolation from specimens

Blood samples were drawn into TEMPUS blood RNA tubes (ABI, USA). RNA was obtained either from whole blood using a Tempus Spin RNA kit (Applied Biosystem (ABI), USA) or from prepared blood with red blood cells removed using Ficoll-Hypaque solution (Sigma, USA) and Trizol (Invitrogen, USA).

Tissues stored at −80°C were subjected to homogenization in an Omni Bead Ruptor12 Homogenizer (Cole-Parmer, USA) in the presence of Trizol (Invitrogen, USA) to isolate total RNA.

### Cell culture, transfection, and RNA extraction

Human normal colon epithelial cells FHC and CRC cells (HCT116, Caco2, LoVo, SW480, SW620) were all obtained from American Type Culture Collection (USA). SW480 and SW620 cells were cultured in L-15 Medium (Gibco, USA) supplemented with 10% fetal bovine serum (FBS, Sigma-Aldrich, USA) in an atmosphere of 100% air. FHC, HCT116, Caco2, and LoVo cells were cultured in RPMI-1640 cell culture medium (Gibco, USA) supplemented with 10% FBS (Sigma-Aldrich, USA) in a humidified incubator containing 5% CO_2_.

HCT 116 and SW620 cells were subjected to transfection. They were seeded in six-well plates to achieve about 80% of the cell confluence. The sequences of mature miR-1180-3p (miR-1180-3p mimic) and negative control of mimic (NC-mimic) were synthesized and purified by Beijing Generaybiotech (Beijing, China). miR-1180-3p mimic or NC-mimic was transfected into HCT 116 and SW620 cells using Lipofectamine 3000 reagent (Invitrogen, USA) as per the instructions.

RNA from cells was extracted via Trizol (Invitrogen) following the user guide from the manufacturer.

### RNA quantification by real-time quantitative PCR (RT-qPCR)

Total RNA was subjected to concentrations evaluation at a NanoDrop instrument (Agilent, USA) to ensure reliability for the subsequent analysis. For testing the level of miR-1180-3p and *COL12A1* mRNA, the total RNA was reversely transcribed using a High Capacity cDNA Archive Kit (ABI, USA). RT-qPCR for miR-1180-3p was accomplished using TaqMan® MiRNA Assays (ABI, USA). RT-qPCR for evaluation of COL12A1 mRNA expression was achieved by Power Up Sybr Master Mix (ABI, USA). The test was performed at an Applied Biosystems 7500 fast Real Time PCR system (ABI, USA). U6 and GAPDH were used as endogenous controls for the relative quantifications of miR-1180-3p and *COL12A1* mRNA respectively. The results were calculated and expressed according to the 2^−ΔΔCt^ method. The paired primer of miR-1180-3p was 5′-CAGAAACAGCCATCCCAGAG-3′ (F) and 5′-GCCTTCAGCAGGATGTCAAT-3′ (R), *COL12A1* was 5′-TGAGGTCTGGGTAAA GGCAA-3′ (F) and 5′-GTATGAGGTCACCGT CCAGG-3′ (R), GAPDH was 5′-TGGGTGTGAACCATGAGAAGT-3′ (F) and 5′-TGAGTCCTTCCACGATACCAA-3′ (R), and U6 was 5′-CTCGCTTCGGCAGCACATATACT-3′ (F) and 5′-ACGCTTCACGAATTTGCGTGTC-3′ (R).

### Conventional tumor markers

An aliquot of each serum sample was subjected to the measurement for CEA, CA125, and CA19-9 levels in our clinical laboratory. Samples were determined by electrochemiluminescence immunoassay at Roche E170 automatic immunity analyzer with kits (Roche, Germany).

### Cell counting kit-8 (CCK-8) proliferation assay

CCK-8 (MedChem Express, USA) assay was used to examine the proliferation of HCT 116 and SW620 cells. In brief, HCT 116 (2000 cells/well) or SW620 (3,000 cells/well) were plated in 96-well plates in 100 μl of RPMI-1640 medium or L-15 Medium (Gibco, USA). Every 24 hours in 72 hours, 10 microliters of the CCK-8 solution were mixed into the indicated well, and the plate was incubated for another two hours at 37°C. Cell numbers were expressed as the absorbance at 450 nm.

### Caspase-3 apoptosis assay

The cell apoptosis was estimated using caspase-3 level via a caspase-3 (active) human ELISA Kit (Invitrogen, USA) as described previously [24]. In brief, transfected HCT 116 and SW620 cells were seeded in 6-well plates with RPMI-1640 medium or L-15 medium, respectively. After 48 hours, the cells were collected and lysed to determine the optical density (OD) at 450 nm with an ELISA microplate reader (BIOBASE, China). The concentration of caspase-3 was calculated by the constructed standard curves.

### Transwell migration and matrigel invasion assays

HCT 116 and SW620 cell migration was evaluated by Costar Transwell inserts (Corning, USA) and invasion by Matrigel Invasion Chambers (BD, USA) [25,26]. For migration assay, transfected cells were serum-starved and then seeded (1× 10^4^ cells per well) onto transwell membranes with serum-free RPMI-1640 medium or L-15 Medium. Lower chambers of companion plates were enriched with 10% FBS medium as a chemoattractant. Incubated for 24 h at 37°C, non-migrated cells on inner surfaces were scrapped, whereas migrated cells were fixed, washed and stained. Five fields were imaged using the Microscopes and Imaging Systems (Leica Microsystems, USA) from each transwell membrane, and the number of migrated cells was counted. As for invasion assays, Matrigel Invasion Chambers was used and performed as migration assay, except for the incubation time was 36 h.

### Bioinformatic analysis

To obtain the potential target gene of miR-1180-3p, three online databases were retrieved: TargetScan Human 7.2 (http://www.targetscan.org/vert_72/), miRDB (http://mirdb.org/), miRWalk (http://mirwalk.umm.uni-heidelberg.de/).

### Assay of luciferase activity

To validate the target gene, HCT 116 or SW620 cells were transfected with dual-luciferase reporters, wide type *COL12A1* with miR-1180-3p 3ʹ-UTR target expression clone (*COL12A1*-WT) and its mutant type (*COL12A1*-MUT) according to the instructions from Promega about pGL4. Following 48 h, HCT 116 or SW620 cells were transfected with miRNA-1180-3p mimic or NC-mimic using Lipofectamine 3000. Luciferase activity assays were performed 48 h after transfection with the use of Luc-Pair™ Duo-Luciferase HS Assay Kit (GeneCopoeia, USA).

### Statistical analysis

Paired t test was used to test the miR-1180-3p difference between CRC tissues and normal tissues adjacent to cancer. Unpaired t test was employed to access the difference of miR-1180-3p in serum and cells between CRC and the healthy. Receiver operating characteristic (ROC) analysis was introduced to compare the diagnostic value among miR-1180-3p, CEA, CA19-9, and CA125. The Kaplan–Meier (K-M) overall survival estimator was used for prognosis value analysis, calculating from the time at diagnosis to the time of death or the end of 5-year follow-up end the survival. Chi-square tests were used to evaluate the correlations between miR-1180-3p level and clinical parameters. The risk ratio of each parameter for death was estimated by Multivariate Cox’s proportional hazard analysis. Statistical significance was assessed at <0.05.

## Results

miR-1180-3p plays important role in cancers. Here, the expression of miR-1180-3p was determined in CRC tissues and cells by RT-qPCR, and showed a downregulated level in CRC. The diagnostic value was evaluated by ROC curves, and miR-1180-3p represented as a potent diagnostic factor in CRC. The prognostic value was accessed by K-M curve and multivariate analyses, identified as a novel prognostic biomarker in CRC. The role of miR-1180-3p in regulating CRC progression was determined based on the cell function such as proliferation, migration and invasion. Furthermore, miR-1180-3p was identified as a molecular regulator of *COL12A1* in CRC.

### Levels of miR-1180-3p decreased in tissues and blood from CRC patients and CRC cells

**Figure 1.**
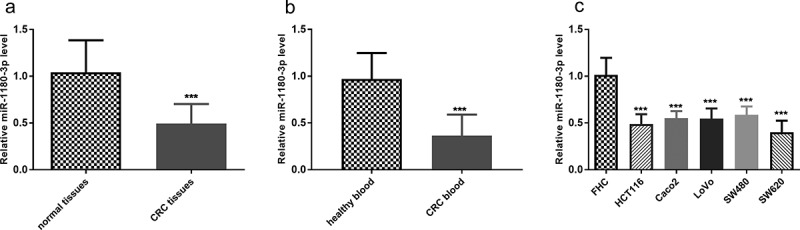
miR-1180-3p level was downregulated in colorectal cancer tissues, blood and cells. (a) The miR-1180-3P expression levels in normal tissue adjacent to cancer and colorectal cancer (CRC) tissues by RT-qPCR. The difference was analyzed by paired t test. (b) The miR-1180-3p expression levels in blood from patients with colorectal cancer (CRC) and healthy subjects by RT-qPCR. The difference was analyzed by unpaired t test. (c) The miR-1180-3P expression levels in colon normal epithelial cell FHC and colorectal cancer cells, HCT116, Caco2, LoVo, SW480, SW620 by RT-qPCR. The difference was analyzed by unpaired t test. ***P < 0.001

The expression of miR-1180-3p in CRC tissues, blood, and cells was determined, along with that in normal ones. By RT-qPCR, miR-1180-3p expression in tumors was lower than the expression in the normal tissues adjacent to the tumor (*P* < 0.001, Figure 1(a)). Lower expression was also observed in blood samples from CRC patients than healthy subjects (*P* < 0.001, Figure 1(b)). Likewise, miR-1180-3p showed a decreased expression in human CRC cells versus that in human normal colon epithelial cell FHC, and HCT116 and SW620 were especially outstanding (*P* < 0.001, Figure 1(c)).

### Evaluation of the discriminatory ability based on the expression of miR-1180-3p in CRC and healthy

**Figure 2.**
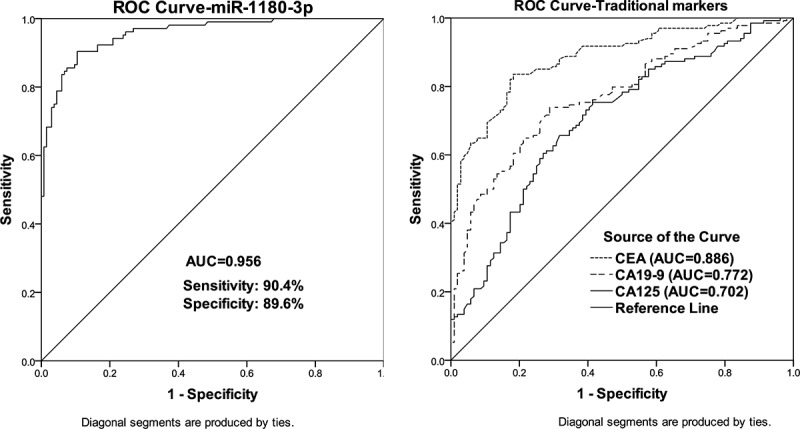
Receiver operating characteristic (ROC) curve analyses of miR-1180-3p, CEA, CA19-9, and CA125 in patients with CRC. (a) ROC curves for miR-1180-3p in patients with CRC. (b) ROC curves for the traditional biomarkers in patients with CRC

For the diagnostic value of miR-1180-3p, CRC patients were divided into a low and high miR-1180-3p level group according to the mean value of miR-1180-3p level in total CRC blood. From Table 1, the low miR-1180-3p expression level in the blood from CRC patients was strongly related to positive vascular invasion (*P* = 0.005) and present lymph node metastasis (*P* = 0.001), and tend to be correlated with the advanced TNM stage (*P* = 0.030). A ROC curve was also generated to access the significance of miR-1180-3p level for CRC diagnosis. As shown in (Figure 2), among the four serum markers, miR-1180-3p had the highest AUC value (0.956) for CRC diagnosis with a sensitivity of 90.4% and specificity of 89.6%, which indicate miR-1180-3p level owned strong discriminatory ability in CRC and healthy.

**Table 1.** Key clinical characteristics and their association with miR-1180-3p expression in blood from CRC patients

Characteristics	Cases (n = 134)	miR-1180-3p expression		*P*
		Low (n = 70)	High (n = 64)	
Age				0.171
≤ 60	69	40	29	
> 60	65	30	35	
Gender				0.156
Female	63	37	26	
Male	71	33	38	
Tumor location				0.501
Left-side colorectal	84	42	42	
Right-side colorectal	50	28	22	
Vascular invasion				0.005
Absent	103	47	56	
Present	31	23	8	
Lymph node metastasis				0.001
Negative	97	42	55	
Positive	37	28	9	
TNM stage				0.030
I–II	88	40	48	
III–IV	46	30	16	
Differentiation				
Well/moderate	75	36	39	0.268
Poor	59	34	25

### miR-1180-3p was positively correlated with overall survival

**Figure 3.**
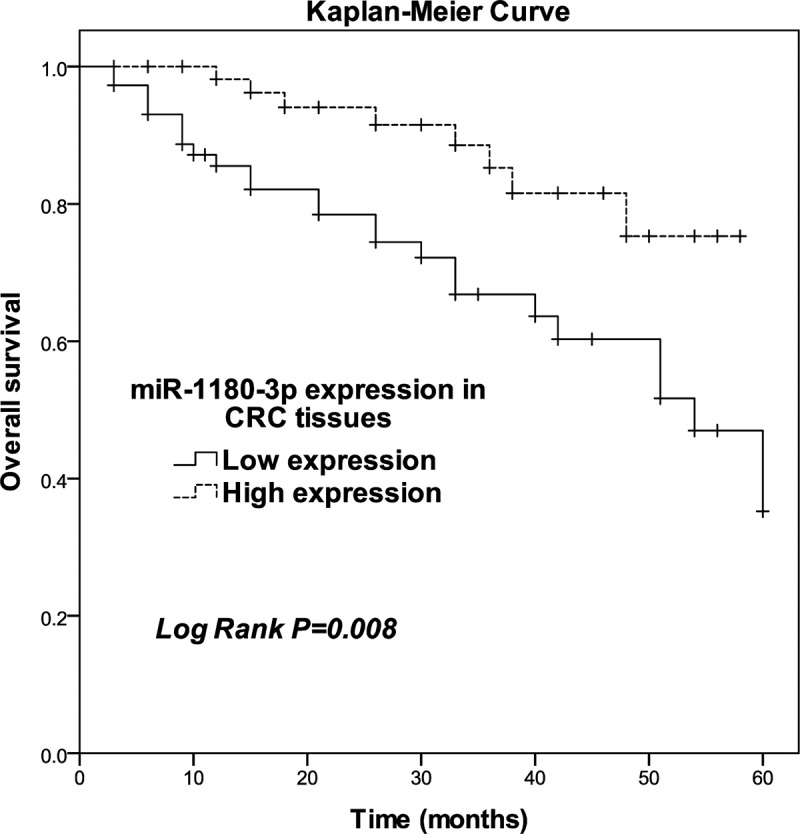
Survival estimates were calculated by Kaplan-Meier analysis. Log-rank test *P* = 0.008

Next, we hypothesized that the miR-1180-3p level is related to the survival of CRC patients. Thus, we assessed the relationship of miR-1180-3p level with overall survival. The patients were classified into low and high expression groups according to the mean level of miR-1180-3p in total of 134 CRC tissues. After K-M curve plotting, we identified low miR-1180-3p expression as being significantly associated with poor overall survival (*P* = 0.008; Figure 3). The result of the chi-square test (Table 2) showed low miR-1180-3p expression level in tissues from CRC patients was correlated with positive vascular invasion (*P* = 0.003), present lymph node metastasis (*P* = 0.001), and advanced TNM stage (*P* = 0.011). After multivariate analyses of potential risk factors of cumulative overall survival, miR-1180-3p was found to be an independent prognostic factor for CRC overall survival (*P* = 0.002, risk ratio: 4.171, 95% CI: 1.656–10.505; Table 3).

**Table 2.** Key clinical characteristics and their association with miR-1180-3p expression in tissues from CRC patients

Characteristics	Cases (n = 134)	miR-1180-3p expression		*P*
		Low (n = 73)	High (n = 61)	
Age				0.237
≤ 60	69	41	28	
> 60	65	32	33	
Gender				0.201
Female	63	38	25	
Male	71	35	36	
Tumor location				0.177
Left-side colorectal	84	42	42	
Right-side colorectal	50	31	19	
Vascular invasion				0.003
Absent	103	49	54	
Present	31	24	7	
Lymph node metastasis				0.001
Negative	97	44	53	
Positive	37	29	8	
TNM stage				0.011
I–II	88	41	47	
III–IV	46	32	14	
Differentiation				
Well/moderate	75	36	39	0.090
Poor	59	37	22

**Table 3.** Multivariate analyses of risk factors for cumulative overall survival in CRC patients

Factors	Multivariate analysis		
	Risk ratio	95% CI	*P*
miR-1180-3p (Low to High)	4.171	1.656–10.505	0.002
Age (> 60 to ≤ 60)	1.177	0.545–2.541	0.678
Gender (Male to Female)	1.225	0.568–2.640	0.605
Tumor location (Right -side colorectal to Left-side colorectal)	1.601	0.763–3.357	0.213
Vascular invasion (Present to Absent)	3.396	1.170–9.860	0.025
Lymph node metastasis (Positive to Negative)	3.658	0.958–13.965	0.058
TNM stage (III–IV to I–II)	3.710	1.188–11.587	0.024
Differentiation (Poor to Well/Moderate)	1.726	0.810–3.675	0.157

### miR-1180-3p overexpression reduced cancer cell proliferation and mobility, but induced apoptosis

**Figure 4.**
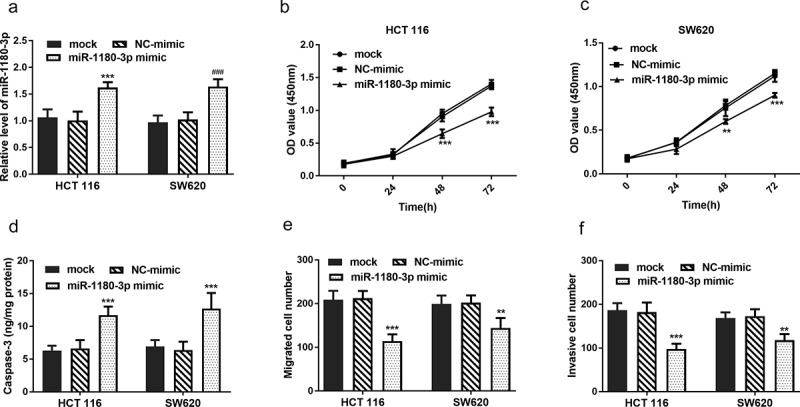
miR-1180-3p overexpression hampered proliferation, migration and invasion in human HCT 116 and SW620 cells. (a) Efficiency of miR-1180-3p overexpression tested by RT-qPCR. (b) and (c) CCK-8 assay was performed to monitor the cell proliferation. (d) The activity of Caspase-3 was detected to indicate cell apoptosis. (e) Transwell assay was used to determine the cell migration. (f) Matrigel assay was employed to detect the cell invasion. Statistical analysis was all performed by two-way ANOVA analysis. **P < 0.01, ***P < 0.001

To clarify the role of miR-1180-3p in cellular function regulation, it was hypothesized that miR-1180-3p may affect cell proliferation, apoptosis, and mobility. To verify these, HCT 116 and SW620 cells were transfected with miR-1180-3p mimics successfully (*P* < 0.001, Figure 4(a)). To test whether miR-1180-3p affects cell proliferation, CCK-8 assay was performed and the results showed overexpression of miR-1180-3p led to a significant suppression on HCT 116 and SW620 cell proliferation (*P* < 0.01, Figure 4(b,c)). To test cell apoptosis alteration, cell apoptosis markers Caspase-3 activity was determined and verified that miR-1180-3p overexpression caused an increase of cell apoptosis (*P* < 0.001, Figure 4(d)). By means of Transwell assay and Matrigel assays, increased miR-1180-3p were observed to inhibiting the mobility of HCT 116 and SW620 cells (*P* < 0.01, Figure 4(e,f)).

### miR-1180-3p directly targets COL12A1

**Figure 5.**
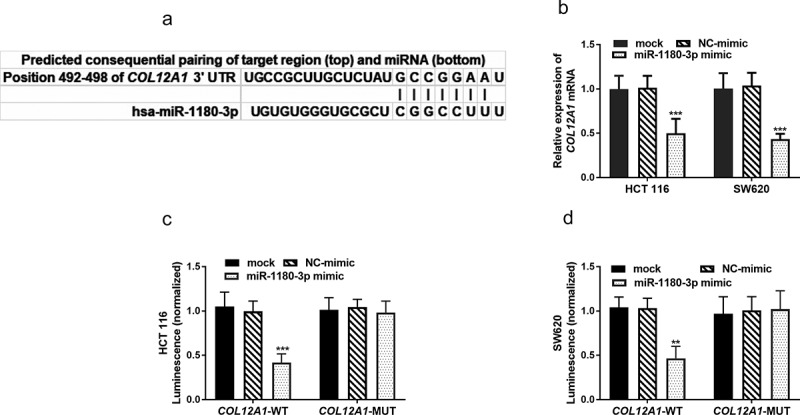
miR-1180-3p directly targets *COL12A1*. (a) Complementarity between miR-1180-3p and the 3′-UTR of *COL12A1*. (b) *COL12A1* mRNA levels were reduced by miR-1180-3p mimic. (c) and (d) Dual luciferase reporter assay to determine the interaction between miR-1180a-3p and *COL12A1* 3′-UTR. Statistical analysis was performed using. Statistical analysis was all performed by two-way ANOVA analysis. **P < 0.01, ***P < 0.001

The search for gene targets of miR-1180-3p rendered *COL12A1* by three online databases collectively, and the binding sites was shown in (Figure 5(a)). Thus, HCT 116 and SW620 cells were transfected with miR-1180-3p mimic and determined the *COL12A1* mRNA expression level using RT-qPCR. The determination showed miR-1180-3p mimic significantly reduced the expression of *COL12A1* mRNA (*P* < 0.001, Figure 5(b)). To further confirm that *COL12A1* is a direct target of miR-1180-3p, HCT 116 and SW620 cells were transfected with luciferase plasmids, which contained either the unaltered or mutated binding site of the miR-1180-3p in *COL12A1* 3′-UTR. A significant reduction of luciferase activity was detected in cells transfected with luciferase plasmids containing wild-type *COL12A1* when compared with the mutated plasmids (*P* < 0.01, Figure 5(c,d)), which confirmed that *COL12A1* is directly targeted by miR-1180-3p.

## Discussion

According to the database from World Health Organization, cancers with 244.6 million Disability-Adjusted Life Years impose the largest worldwide healthy burden both in men and women [27]. An early diagnosis of CRC along with effective and reliable prognostic factors would greatly reduce the mortality rate of CRC [28]. Evaluation of the specific miRNAs’ expression levels in clinical tumor tissue or blood samples may be useful for detecting cancer and predicting the cancer prognosis [29]. In this current study, the expression level of miR-1180-3p was detected in CRC tissues and blood samples intending to figure out its clinical value in CRC diagnosis and prognosis and investigate its role in cell function and the potential mechanism.

By RT-qPCR, a lower expression level of miR-1180-3p was observed in CRC tissues, blood and cells compared with the normal samples. These results confirm Gmerek’s study that demonstrated the downregulation of miR-1180-3p in CRC [22]. Besides, this study showed the reduced expressions of miR-1180-3p in blood and tissues were both related to unfavorable clinical factors, such as positive vascular invasion, positive lymph node metastasis, and advanced TNM stage. Using ROC curve analysis of expression level in blood samples, miR-1180-3p was verified to owning the discriminating ability between CRC and healthy participants. In previous studies, miR-1180-3p has showed its diagnostic value in melanoma, gastric cancer, and early hepatocellular carcinoma [21,30,31]. This finding would shed light on the development of miR-1180-3p as a new noninvasive diagnostic marker for CRC. It was formerly demonstrated that miR-1180-3p was associated with a poor prognosis in hepatocellular carcinoma [32]. As for the estimating ability of miR-1180-3p in CRC prognosis, K-M curve and multi-variate analysis provided evidence for miR-1180-3p significantly associating with overall survival and as an independent prognostic factor. Combined with the above, miR-1180-3p has great potentiality of being a diagnostic and prognostic biomarker for patients with CRC.

Given that miR-1180-3p has been known as a suppressor in bladder cancer and pancreatic cancer [33,34], it was hypothesized that miR-1180-3p may affect CRC cell proliferation, mobility, and apoptosis. Through the experiment of CCK-8, CRC cell proliferation was monitored to be inhibited by overexpression of miR-1180-3p. By means of Transwell assay, cell mobility including migration and invasion was impeded by upregulation of miR-1180-3p. Cell apoptosis can be induced by miR-1180-3p upregulating represented by the activity of Caspase-3. Based on these results, it is presumed that miR-1180-3p may be a tumor suppressor in CRC, which inhibits cell growth and mobility but induces cell apoptosis.

MiRNAs are known for mediating their target genes’ post-transcriptional silencing by targeting the 3ʹ-UTR of corresponding mRNA [35]. For instance, miR-145-5p could inhibit PLD5 resulting in downregulation of cell proliferation and metastasis in prostate cancer [36]. miR-1180-3p has been reported involving in the regulation of C11of54 in hepatocellular carcinoma and *ST3GAL4* in cutaneous melanoma [30,37]. The search for gene targets of miR-1180-3p rendered *COL12A1* by three online databases. *COL12A1* has been reported to be upregulated in CRC and associated with a poor prognosis of CRC [38]. Further, *COL12A1* mRNA level showed an expected reduction when cells were transfected with miR-1180-3p mimic. In addition, the in-silico prediction was verified by cloning 3ʹ-UTR of *COL12A1* gene into the 3ʹ-UTR regions of a reporter vector, followed by co-transfection with miR-1180-3p mimic. Assay of luciferase activity was reduced by a wide type of target 3ʹ-UTR, but the effect no longer existed after the binding sites were mutated. Therefore, miR-1180-3p may act as a tumor-suppressive effect by directly targeting *COL12A1*.

## Conclusion

Taken together, our findings uncover the important clinical significance of miR-1180-3p in the diagnosis and prediction of prognosis for patients with CRC. Overexpression of miR-1180-3p may be a tumor suppressor, which inhibits cell growth and mobility but induces cell apoptosis by targeting *COL12A1*. This study provides novel insights into the development of a new biomarker for CRC diagnosis and prognosis, and also a new molecular therapeutic target.

## Highlights

- miR-1180-3p is downregulated in colorectal cancer.
- miR-1180-3p has diagnostic and prognostic value in colorectal cancer.
- miR-1180-3p might function as a suppressor in colorectal cancer.
- *COL12A1* is target gene of miR-1180-3p in colorectal cancer.
